# Student feedback about The Skeptic Doctor, a module on pharmaceutical promotion

**DOI:** 10.3352/jeehp.2011.8.11

**Published:** 2011-11-30

**Authors:** P. Ravi Shankar, Kundan K. Singh, Rano M. Piryani

**Affiliations:** KIST Medical College, Lalitpur, Kathmandu, Nepal.

**Keywords:** Learning, Marketing, Students, medical, Nepal, Problem-based learning

## Abstract

Pharmaceutical promotion is an integral part of modern medical practice. Surveys show that medical students have a positive attitude towards promotion. Pharmaceutical promotion is not adequately taught in medical schools. A module based on the manual produced by Health Action International was conducted for second year medical students at KIST Medical College, Lalitpur, Nepal. Student feedback on various aspects of the module was obtained using a semi-structured questionnaire. Eighty-six of the 100 students (86%) provided feedback about the module. Forty-five (52.3%) were female and 39 (45.3%) were male. Participant feedback about the module was positive. Small group work and role plays were appreciated, and the ratings of the module and the manual were satisfactory. Respondents felt pharmaceutical promotion will play an important role in their future practice and that the module prepared them to respond appropriately to promotion and select and use medicines properly. The module further developed on issues covered during pharmacology practical and majority felt the module was of relevance to Nepal. Students appreciated the module though there were suggestions for improvement. The module should be considered during the years of clinical training (third and fourth years) and internship and in other medical schools.

## INTRODUCTION

Pharmaceutical promotion is a ubiquitous aspect of modern medicine [1], which involves and targets essentially all elements of medicine and healthcare. Students begin to have contact with the pharmaceutical industry early in their career [2]. Medical students and postgraduate trainees may have positive attitudes towards pharmaceutical promotion [3], frequently engage in contacts with the industry [4], and deny they are influenced by promotion [3]. A similar result was shown by a review carried out in the US [5].

A national survey conducted in the US concluded that student experiences and attitudes suggest that as a group they may be at risk of unrecognized influence by marketing efforts by the industry [6]. A Canadian survey showed a large majority of medical students were not opposed to interacting with the industry in medical school and felt comfortable accepting industry gifts [7]. A similar result was noted in a survey in India where 95% believed that information provided by medical representatives (MRs) was reliable and 75% opined confirmation of the claims was not required [8]. In Nepal, pharmaceutical companies strongly promote their medicines to doctors, and most hospitals allow free and unrestricted access of MRs to doctors [9]. In 2002, the American Medical Students Association (AMSA) launched a nationwide PharmFree campaign encouraging doctors in training to seek out objective and unbiased sources of medicine information [10].

At the University of Chicago in the US, interested internal medicine residents participated in a controlled intervention across the three years of residency [11]. Residents' perceptions towards gifts and interactions with the industry changed modestly after the educational intervention. At the Manipal College of Medical Sciences, Pokhara, Nepal, an educational initiative on pharmaceutical promotion was conducted for second year undergraduate medical (MBBS) students [9]. Critical analysis of promotional material and drug advertisements and role plays of interaction between MRs and doctors [12] were among the activities conducted. At KIST Medical College (KISTMC), Lalitpur, Nepal, students learn to critically analyze drug advertisements and promotional material, learn to optimize time spent with MRs, and become familiar with sources of medicine information [13]. Critical analysis of drug advertisements and promotional material is assessed during the pharmacology practical examination [14]. Recently, the World Health Organization (WHO) and Health Action International (HAI) have produced a manual on pharmaceutical promotion that aims to enable medical and pharmacy professionals to reconsider their central role as a target for pharmaceutical marketing and prepare them to respond appropriately. A module on promotion based on the manual was designed and conducted for second year MBBS students of KISTMC. The module was designed to add to and build upon the teaching about pharmaceutical promotion during pharmacology practical sessions. The present study was conducted with the following objectives:

1. Obtain participant feedback about 'The Skeptic Doctor', the module on pharmaceutical promotion
2. Understand problems and difficulties during the module, and
3. Obtain suggestions for further improving the module in the future.

## MATERIALS AND METHODS

The module entitled 'The Skeptic Doctor' was held on Mondays during early clinical exposure from April to early August of 2011. The 100 students of the 2009 intake were divided into two batches of 50 students each. Each batch was further subdivided into five small groups of 10 students. The sessions were held on alternate Mondays for a particular batch from 8.05 to 9.10 am and used facilitator presentations, case scenarios, brainstorming sessions, group activities, and role plays to explore different aspects of pharmaceutical promotion. Two of the authors (PRS and RMP) acted as facilitators for the small group sessions. For certain sessions, students acted as facilitators under our guidance and supervision. At the same time, the other batch completed assignments and group exercises based on the manual under the supervision and guidance of the author (KKS).

The sessions conducted were 'Promotion of medicines and public health', 'Techniques that influence the use of medicines', 'Analyzing pharmaceutical advertisements in medical journals', 'Pharmaceutical sales representatives', 'Promotion to consumers', 'Students and the pharmaceutical industry', and 'Using unbiased prescribing information'. The topics were selected based on the manual and considering their present and future importance in medical practice. The activities were to 'Create a poster advertisement for a new antibiotic belonging to the group Macrolides and targeted at doctors', 'Create a 2 minute radio advertisement for a sleeping pill targeted at the lay public', analyze advertisements in medical journals and industry promotional material using criteria mentioned in the manual, and analyze videos of television advertisements of a fairness cream, antidepressants, hypnotics, and a blog site to promote medicines. Students critically analyzed and explored offers by the pharmaceutical industry to promote different student events and offers of free textbooks and other equipment. They also developed criteria to assess the quality of health websites on the internet, which was followed by a discussion and facilitator input. Ethical issues concerning the relation between pharmaceutical companies and doctors were also analyzed using role plays.

Participant feedback regarding the module was obtained using a semi-structured questionnaire in mid-August, 2011. The questionnaire was tested for readability and ease of understanding among two faculty members and two third year students. Participants were explained the aims and objectives of the study and invited to participate. Written informed consent was obtained. The study was approved by the Institutional Review Board of KIST Medical College. The demographic information collected included gender and method of financing of medical education. Participants were asked to provide two overall comments about the module, and their comments about group work and role plays used in the module. The appropriateness of the scenarios used, suggestions for further improving the module, and reasons why future doctors should know about promotion were solicited.

Participants were asked to rate on a scale of 1 to 10 (with 1 being least and 10 being the maximum) their perception regarding the effectiveness of the module. On a scale of 1 to 5 (1 being the least and 5 being the maximum), they rated their enjoyment of the module and the manual on which the module was based.

The participant responses were collected and grouped together and the number of respondents stating each response noted. Responses were either quoted verbatim by the authors or paraphrased in the Results. For the three ratings, the mean score was calculated and compared among subgroups using an independent samples t-test. A P-value less than 0.05 was taken as statistically significant.

## RESULTS

Eighty-six of the 100 second year students (86%) gave their feedback about the module. Forty-five (52.3%) were female and 39 (45.3%) male. Two respondents did not indicate their gender. There were nine scholarship students and 76 students (88.4%) were self-financing, with one not mentioning this information.

Table 1 shows the overall comments of student respondents about the module. Other comments were as follows: certain group members did not participate as expected; students learned to critically analyze drug promotional material and learnt to assess internet health information. Two respondents stated the module was a bit boring at times.

Respondents' comments about group work, which was an important part of the module, were that the group in which they were working was cooperative (16 respondents, 18.6%), in the group each member had freedom to express his/her views (15 respondents, 17.4%), and the group work increased group participation (15 respondents, 17.4%). Table 2 shows the participants' perception about how role plays helped in realizing the objectives of the module. In addition participants felt role plays helped in addressing many issues in a short period of time, provided visual and auditory inputs, and helped solve problems in practice.

Participants were not aware about similar modules in other medical schools though the Institute of Medicine and Patan Academy of Health Sciences have stated that they conduct some sessions on promotion. The mean±SD effectiveness of the module according to the respondents was 7.75±0.88 and the mean±SD enjoyment score was 3.53±0.72. Eighty-two of the 86 respondents felt the scenarios covered during the small group activities and role plays were appropriate. Among the reasons mentioned were that the scenarios covered future problems in students' medical practice and also addressed possible ways of tackling these (19 respondents, 22.1%), the scenarios represented the real situation of the country (7 respondents, 8.1%) and the scenarios were based on session objectives (6 respondents, 7%). Other reasons mentioned were that the scenarios promoted active learning and enhanced creativity. Table 3 lists respondents' suggestions for further improving the module. Other suggestions were to make the module compulsory and include it in the syllabus, have more facilitators, conduct the module for future cohorts, have more material from Nepal, and simplify the language and explain the statistics more.

The mean±SD rating of the manual was 3.81±0.64. Students felt the manual was informative and enjoyable and provided basic knowledge about promotion that every doctor should know. They felt more scenarios from South Asia and Nepal should be included. Twenty-six respondents (30.2%) experienced difficulties during the module. Among the difficulties were new terminology and phrases, difficulty in understanding certain topics, and problems with statistics, too little time for the sessions, and difficulty in analyzing long articles during the assignments. Fifty-seven respondents did not report any problems.

Table 4 shows respondents' opinions about why future doctors should learn about pharmaceutical promotion. Regarding the application of what they learned from the module, 29 respondents stated they would deal in a better manner with MRs, 19 stated they would assess promotion more carefully, 12 said they would be more careful in choosing drugs, and 7 stated they would be more patient-centered and put the patient first in their medical practice. Students were confident about utilizing the skills learned in future industry interactions.

The participants felt the module linked up with and built on issues covered during pharmacology small group practical sessions. The module added to the information provided, students were able to apply theoretical knowledge practically, and had a better idea of practical scenarios they might encounter in the future. Fifty-three students (61.6%) stated they were confident about using statistics to interpret clinical research and evidence presented in the scientific literature while 25 (29%) stated they were not confident and wanted more classes in statistics and using statistics to interpret clinical trial data. Seventy-one students (82.5%) wanted a similar module in the future.

Among the other comments were 'thank you for conducting the programme', 'the sessions were most effective' and 'I think the module should be compulsory in every medical school. After this module I learnt how much drug companies spend on doctors, MRs and how this is affecting patients' health.' Another stated 'It was a wonderful experience and probably a unique one.'

## DISCUSSION

Participant feedback about the module was positive. Small group work and role plays were appreciated and the ratings of the module and the manual were satisfactory. The respondents felt pharmaceutical promotion will play an important role in their future practice and the module prepared them to respond appropriately to promotion and select and use medicines properly. The module further expanded on issues covered during the pharmacology practical and majority felt the module was of relevance to Nepal.

The module was not a formal part of the curriculum and was not assessed. Attendance was a problem during certain sessions though overall attendance during the module was over 70%. The learning methods used were similar to those employed during the Medical Humanities module. Small group work, brainstorming sessions, and role plays were used during this module also. This was also the first time we conducted the module, and hopefully we will improve it in future.

A survey of educational initiatives for medical and pharmacy students about pharmaceutical promotion showed that over 60% of institutions worldwide had used small group discussions in tutorials or workshops [15]. Response to case scenarios was used in about 25% of institutions while role playing was used in only 10%. Most schools had a one or two hour lecture on the topic. At the University of California Los Angeles in the US, a university pharmacist played the role of a pharmaceutical representative (PR) promoting a non-sedating antihistamine before small groups of students and faculty [16]. Students were unaware that these PRs were actually university pharmacists. They were encouraged to ask questions after the presentation. Student attitudes towards promotion were favorably impacted by the exercise.

We use role plays to teach students to optimize the time spent with MRs [13]. In the US, an innovative workshop used role plays with pharmaceutical representatives to demonstrate appropriate and inappropriate interaction strategies [17]. Using former MRs or even working MRs would be a good strategy to sensitize students to the techniques used but might be difficult to organize. This 'initial' module based on the manual aimed to generate evidence of acceptability and effectiveness so that a case can be made for introducing a similar module in other medical schools and eventually including it in the curriculum of different universities in Nepal. Our students do not usually read journal articles. Journal articles (including original articles, and different types of reviews are important independent information sources for doctors and obtaining relevant information from these sources have been mentioned in the HAI-WHO manual. As several respondents noted, the module may be more effective during the final year or even during the internship when students have more interactions with MRs and are more exposed to promotion.

Student rating of the manual was positive. The manual is well designed and organized and easy to read and understand. Certain parts may be difficult. The major problem is the paucity of studies from South Asia and Nepal. This may be because data from this region and other developing nations are lacking and put a major responsibility on the present authors and others from developing countries to generate this data for the module. At the end of the module many respondents stated that they will be able to deal in a better manner with MRs. Resistance to misleading promotion can be improved by making physicians understand that they are vulnerable to the influence of promotion [18].

Role modeling by clinician teachers and other doctors and their attitudes towards promotion play an important role in determining student attitudes towards the industry and promotion [19]. In our institution, the Medicine and Therapeutics Committee regulates interactions between prescribers and MRs [20], and many clinicians are aware of the negative impact of promotion on prescribing behavior.

The strengths of the study were the high response rate and enthusiastic response of the students who participated in the module. The study had limitations. Student feedback was obtained using a questionnaire and was not triangulated with information from other sources. The questionnaire was developed by the authors and was not validated. The students may have had difficulty understanding certain questions. This could be because English the language of instruction is not the first language of the students and certain students were educated in the vernacular medium in school. Also the questionnaire was not validated and revised.

## CONCLUSION

The authors conducted a module on pharmaceutical promotion using available resources and the Health Action International manual. Students appreciated the module, and they felt it prepared them for issues they will encounter in future practice. Improvements are required and will be carried out in the future. The module could be conducted during the clinical years of training and internship. The module could also be considered for inclusion in other medical schools within and outside the country.

## Figures and Tables

**Table 1:**
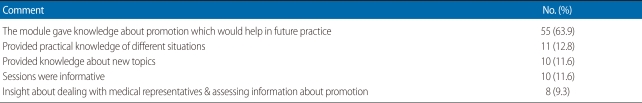
Common overall comments of student respondents about the module (n=86)

**Table 2:**
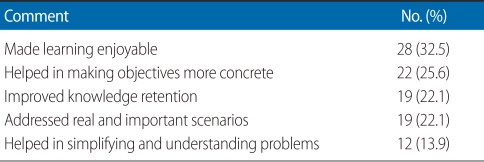
Participants perception of how role plays helped in realizing module objectives

**Table 3:**
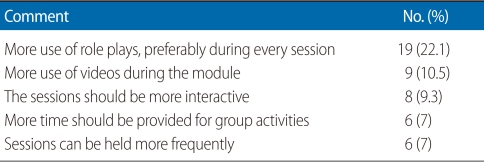
Common suggestions for further improving the module in the future (n=86)

**Table 4:**
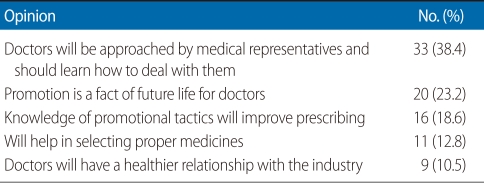
Respondents' opinion about why future doctors should learn about promotion (n=86)
